# Supplementary material for: Oxidative Stress-Induced Misfolding and Inclusion Formation of Nrf2 and Keap1
Source: Antioxidants (Basel). 2022 Jan 27;11(2):243. doi: 10.3390/antiox11020243 (PMC8868093; doi:10.3390/antiox11020243)
Supplement: Supplementary file 1 [file antioxidants-11-00243-s001.zip › antioxidants-1514226-supplementary.pdf]

Supplementary Figures

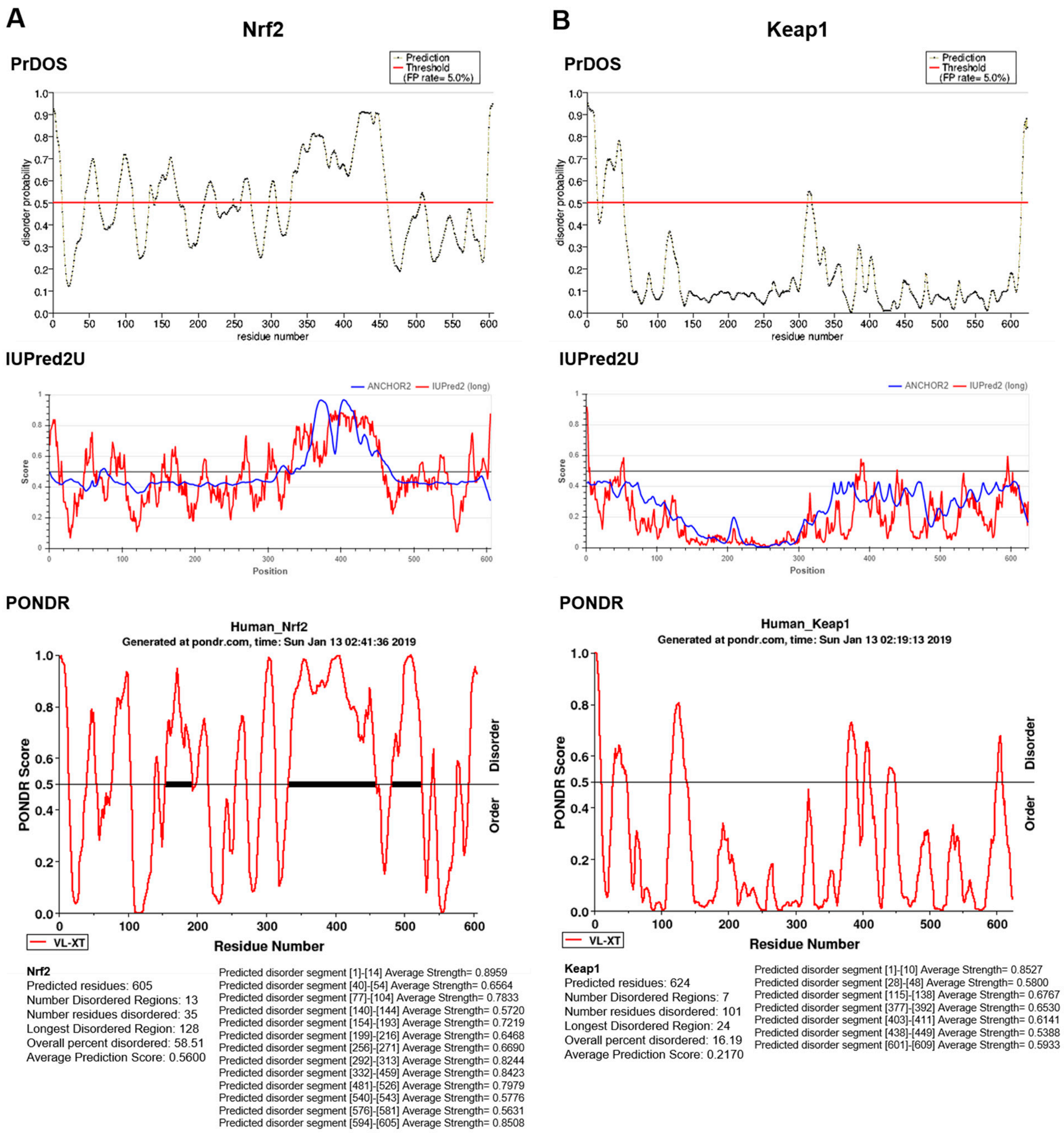

Figure S1: Individual disordered profile plots for PrDOS, IUPred2U, and PONDR. Disorder probability prediction graphs for Nrf2 and Keap1 are shown for all three prediction algorithms. A score of >0.5 predicts disorder and a score of <0.5 predicts order.

Table S1: The 15 species observed in cysteine analysis studies. The species name, specific name, and UniProt code are shown.

	Species	Specific Name	UniProt Code
**Nrf2**	Human	*Homo sapiens*	Q16236
	Chimpanzee	*Pan troglodytes*	H2RAX5
	Orangutan	*Pongo abelii*	H2P7Y6
	Rhesus macaque	*Macaca mulatta*	F7GPD8
	Marmoset	*Callithrix jacchus*	F7CLI8
	Galago	*Otolemur garnettii*	H0Y129
	Mouse	*Mus musculus*	Q60795
	Rat	*Rattus norvegicus*	O54968
	Golden hamster	*Mesocricetus auratus*	A0A1U7QFW3
	Rabbit	*Oryctolagus cuniculus*	G1SEJ1
	Cow	*Bos taurus*	Q5NUA6
	Bat	*Myotis lucifugus*	G1P184
	Elephant	*Loxodonta africana*	G3TGN3
	Chicken	*Gallus gallus*	F1P315
	Zebrafish	*Danio rerio*	Q7ZVI2
**Keap1**	Human	*Homo sapiens*	Q14145
	Chimpanzee	*Pan troglodytes*	H2QFB9
	Orangutan	*Pongo abelii*	Q5R774
	Rhesus macaque	*Macaca mulatta*	G7NL03
	Marmoset	*Callithrix jacchus*	F7HDW0
	Galago	*Otolemur garnettii*	H0X799
	Mouse	*Mus musculus*	Q9Z2X8
	Golden hamster	*Mesocricetus auratus*	A0A1U7R3C2
	Rat	*Rattus norvegicus*	P57790
	Rabbit	*Oryctolagus cuniculus*	G1SFF4
	Cow	*Bos taurus*	A7MBG4
	Bat	*Myotis lucifugus*	G1PRL8
	Elephant	*Loxodonta africana*	G3TJS6
	Chicken	*Gallus gallus*	Q5ZL67
	Zebrafish	*Danio rerio*	E7FB56

Table S2: Yeast oxidative stress gene deletion strains used in this study. The gene name, protein name, function (obtained from UniProt), and UniProt code are shown.

Gene	Protein	**Function** (UniProt Consortium)	UniProt Code
BTN2	Protein BTN2	V-SNARE binding protein that facilitates specific protein retrieval from a late endosome to the Golgi. Modulates the rate of arginine uptake. Involved in pH homeostasis.	P53286
CTA1	Peroxisomal catalase A	Occurs in almost all aerobically respiring organisms and serves to protect cells from the toxic effects of hydrogen peroxide.	P15202
GLR1	Glutathione reductase	Maintains high levels of reduced glutathione in the cytosol.	P41921
SOD1	Superoxide dismutase [Cu-Zn]	Destroys radicals that are normally produced within the cells and which are toxic to biological systems.	P00445
SOD2	Superoxide dismutase	Destroys radicals that are normally produced within the cells and which are toxic to biological systems.	S4VPL7
UBI4	Polyubiquitin	Becomes conjugated to proteins, marking them for selective degradation via the ubiquitin-26S proteasome system.	P0CG63
YAP1	AP-1-like transcription factor YAP1	Transcription activator involved in oxidative stress response and redox homeostasis. Regulates the transcription of genes encoding antioxidant enzymes and components of thiol-reducing pathways.	P19880
SNO4	Probable glutathione-independent glyoxalase SNO4	Catalyzes the conversion of methylglyoxal (MG) to D-lactate in a single glutathione (GSH)-independent step. May play a role in detoxifying endogenously produced glyoxals. Involved in protection against reactive oxygen species (ROS).	Q04902
SRX1	Sulfiredoxin	Contributes to oxidative stress resistance by reducing cysteine-sulfinic acid formed under exposure to oxidants in the peroxiredoxin TSA1. May catalyze the reduction in a multi-step process by acting both as a specific phosphotransferase and as thioltransferase.	P36077
TSA1	Peroxiredoxin TSA1	Thiol-specific peroxidase catalyzing the reduction of hydrogen peroxide and organic hydroperoxides to water and alcohols, respectively.	P34760
TSA2	Peroxiredoxin TSA2	Thiol-specific peroxidase catalyzing the reduction of hydrogen peroxide and organic hydroperoxides to water and alcohols, respectively.	Q04120
TRX1	Thioredoxin-1	Participates as a hydrogen donor in redox reactions through the reversible oxidation of its active center dithiol to a disulfide, accompanied by the transfer of 2 electrons and 2 protons.	P22217
TRX2	Thioredoxin-2	Participates as a hydrogen donor in redox reactions through the reversible oxidation of its active center dithiol to a disulfide, accompanied by the transfer of 2 electrons and 2 protons.	P22803

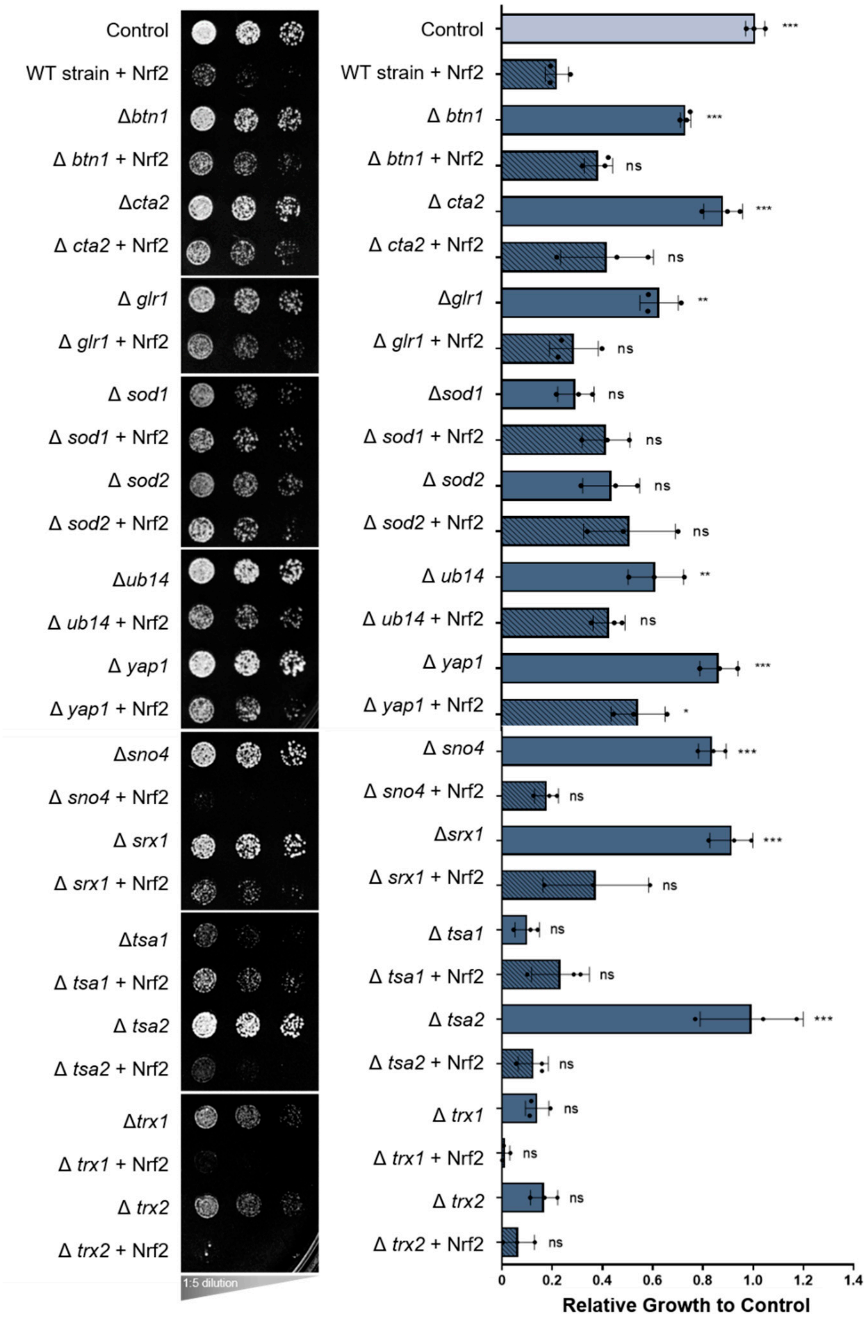

Figure S2: Full panel of growth assays for Nrf2 expression in yeast oxidative stress deletion strains. Growth was quantified relative to control. Means derived from three biological replicates were used during analysis. Means were analyzed using one-way ANOVA followed by Tukey's post hoc test. Data are expressed as mean ± SD. $p<0.05$ was considered statistically significant; *$p<0.05$, ** $p<0.01$, *** $p<0.001$.

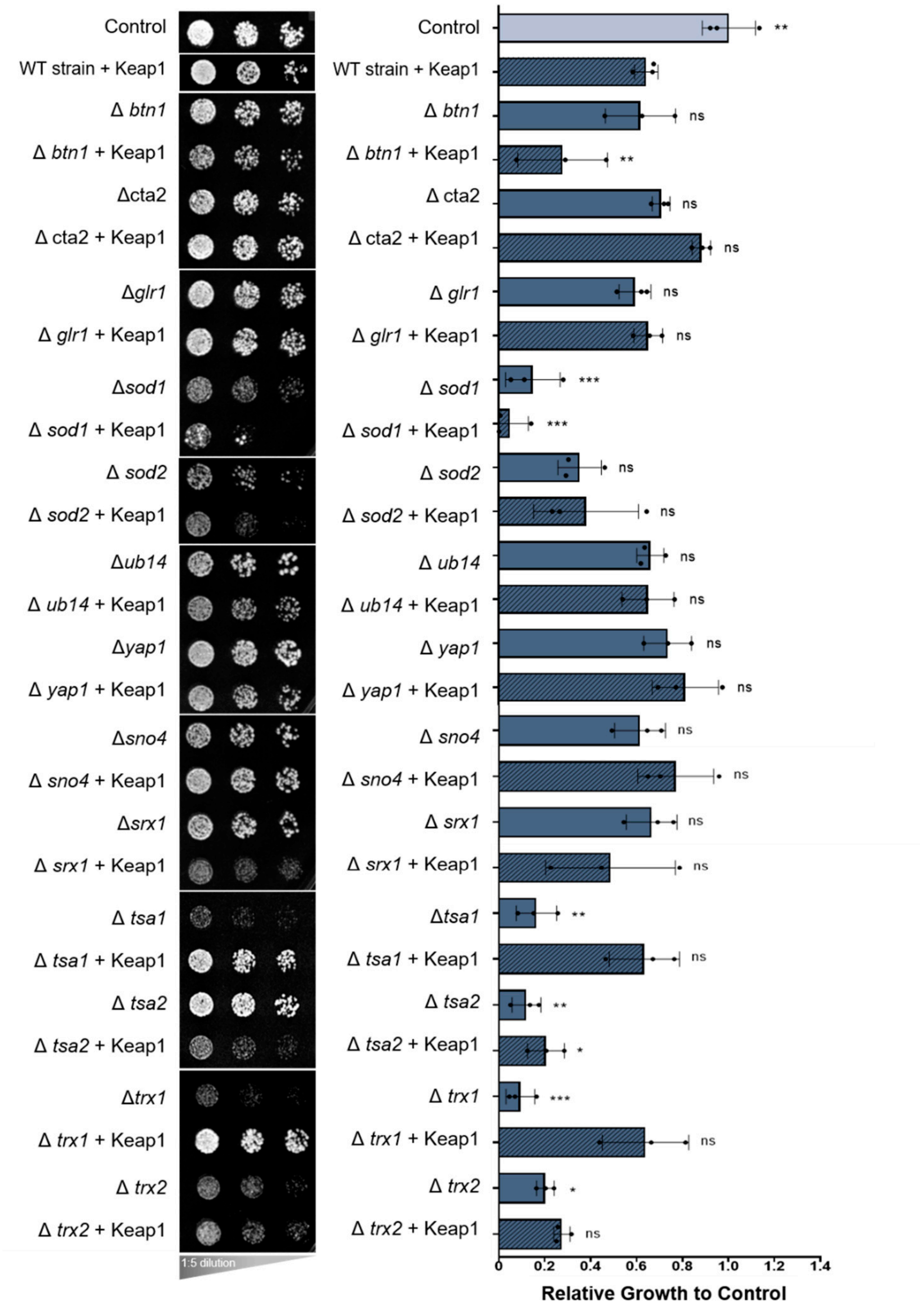

Figure S3: Full panel of growth assays for Keap1 expression in yeast oxidative stress deletion strains. Growth was quantified relative to control. Means derived from three biological replicates were used during analysis. Means were analyzed using one-way ANOVA followed by Tukey's post hoc test. Data are expressed as mean ± SD. $p<0.05$ was considered statistically significant; * $p<0.05$, ** $p<0.01$, *** $p<0.001$.

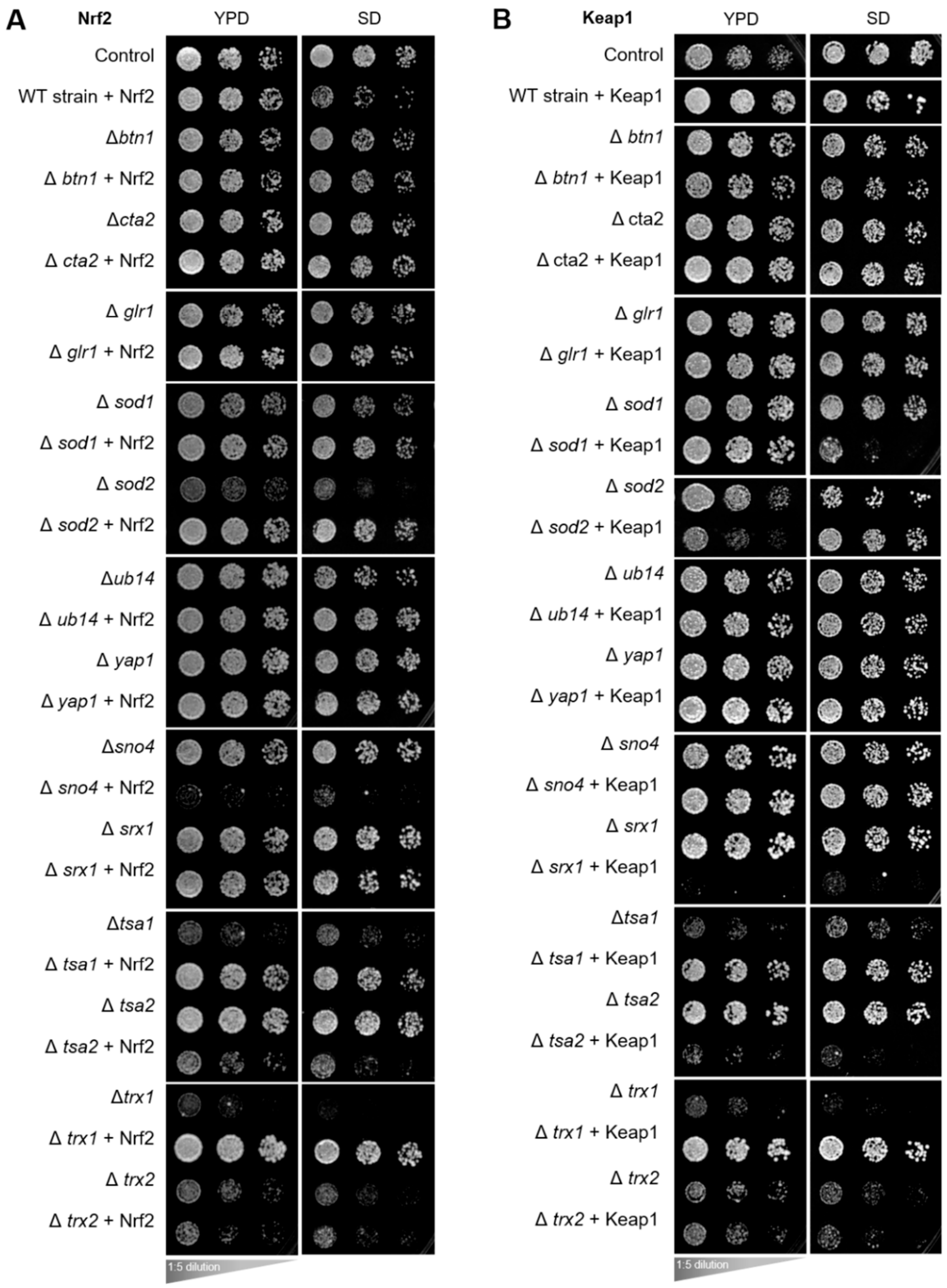

Figure S4: Growth assay control plates for all yeast oxidative stress deletion studies. Yeast- extract-peptone-dextrose (YPD) and selective dextrose (SD) control plates are shown for **(A)** Nrf2 and **(B)** Keap1 experiments. Note that some deletions strains harbour an inherent toxic growth phenotype observed even on control media.

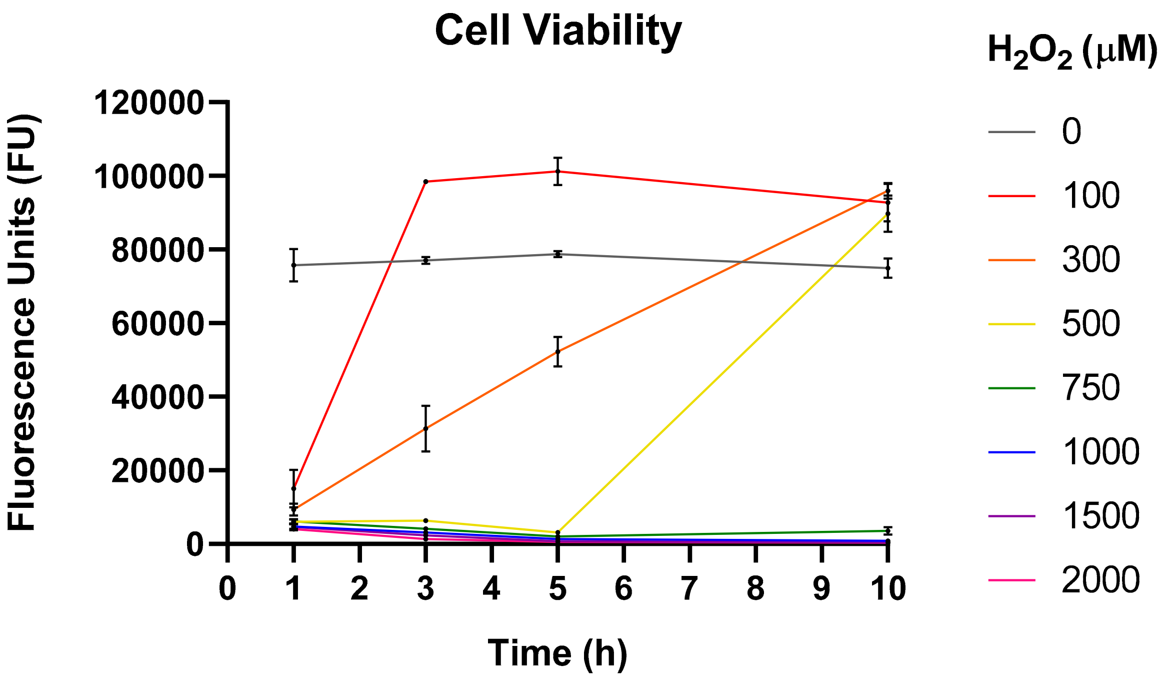

Figure S5: Optimization of hydrogen peroxide treatment concentration and duration. Non-transfected HeLa cells were treated with various concentrations of hydrogen peroxide for 1, 3, 5, and 10 h and cell viability was assessed (measured by ATP levels which indicates the presence of metabolically active cells).

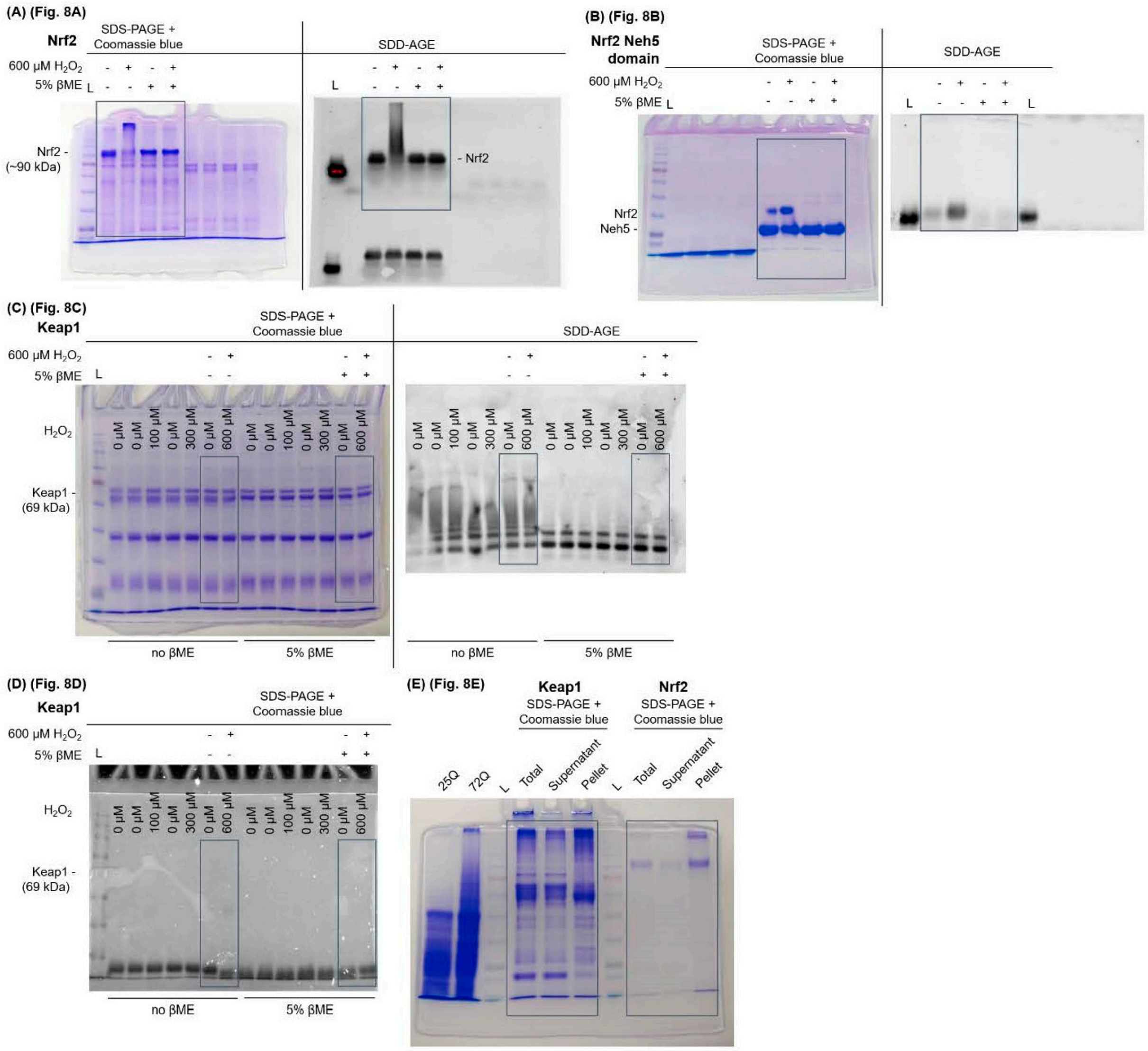

Figure S6: Full uncropped and unadjusted blots for all Coomassie blue and SDD-AGE experiments in Figure 8. (**A–E**) Nrf2 or Keap1 purified protein or protein domains were treated with H2O2 ± βME for 30 mins.
